# Quality indicators for Bariatric Surgery 2004 to 2012: example from Florida

**DOI:** 10.1186/1472-6963-14-S2-P112

**Published:** 2014-07-07

**Authors:** Annie N Simpson, Emily E Johnson, Kit N Simpson

**Affiliations:** 1 Dept. of Healthcare Leadership and Management. Medical University of South Carolina, Charleston, SC, USA; 2 Dept. of Health Science and Research. Medical University of South Carolina, Charleston, SC, USA

## Background

Bariatric surgery is a rapidly diffusing innovation in the field of weight loss. We would expect improvement in quality indicators as more surgeries are performed. However, it is not known if this “practice makes perfect” trend holds for bariatric surgery. We examine three quality indicators for bariatric surgery: rate of surgical complications, death rate and rate of readmissions to a hospital within 30 days from discharge.

## Materials and methods

All elective hospital admissions with a procedure code for bariatric surgery were extracted from the HCUP State Data for Florida for the years 2004 through to 2012. A total of 57,533 surgical admissions were identified (Index admissions). Any admission within 30 days of discharge from the Index admission, regardless of reason for the readmission, was noted as a 30 day readmission (30DR). Admissions with ICD-9 diagnosis codes for surgical misadventure, foreign object, surgical contamination, instrument failure, persistent fistula, shock and hemorrhage were coded as having a complication.

## Results

The mean age of patients was 47.3 years (SD 13.6). The majority of patients were female (74%), of white race (68.4%) with 14.3% black race and 14.4% Hispanic. A diagnosis of diabetes was present for 32% and 87% had a diagnosis code of morbid obesity. The most common surgical approaches were laparoscopic roux-en-y (LREY) 43%, roux-en-y (REY) 22%, and laparoscopic adjustable gastric band (LAGB) 17%. One percent of patients had a percutaneous endoscopic gastrostomy (PEG) tube at the time of surgery.

The overall complication rate was 0.92%, range 0.97% in 2004 to 0.69% in 2012 (p=0.1050). Complications ranged from LSG=0.33% to OSG=2.05% (p<0.0001), after controlling for year, age, sex, race and diabetes. The death rate during the Index admission was 0.92% and did not change over time (p=0.09). Complications during surgery increased the risk of death nearly 3 fold.

Risk of 30DR for bariatric surgery declined from 10.5% in 2004 to 8.4% in 2012 (p<0.0001). The odds of a 30DR decreased by 3.3% per year, after controlling for patient year, age, sex, black race, Hispanic ethnicity, and the presence of a diagnosis of diabetes or morbid obesity. The adjusted odds of 30DR was closely associated with the type of surgery (P<0.0001). The unadjusted 30DR rates ranged from 3.4% for LAGB to 14.1% for REY. Patients with complications had a 76% (p<0.0001) increased risk of 30DR.

## Conclusions

Quality indicators change over time in a complex manner affected by procedure choice, patient characteristics and medical errors.

